# Development aid contracts database: World Bank, Inter-American Development Bank, and EuropeAid

**DOI:** 10.1016/j.dib.2022.108121

**Published:** 2022-04-02

**Authors:** Mihály Fazekas, Aly Abdou, Yuliia Kazmina, Nóra Regős

**Affiliations:** a Central European University, Vienna, Austria; b Government Transparency Insitute, Budapest, Hungary; c University of Amsterdam, Amsterdam, the Netherlands

**Keywords:** Development aid, Public procurement, Contracts, World Bank, Inter-American Development Bank, EuropeAid

## Abstract

This article presents a global database of government contracts funded by the World Bank, Inter-American Development Bank and EuropeAid, principally from the years 2000-2017. The contract-level data were directly collected from the official contract publication sites of these organisations using webscraping methods. While the source publication formats are diverse both over time and across publishers, we standardized and harmonized the datasets so that they can be analysed jointly. The datasets contain key information on the contracting parties (e.g. buyer and supplier names) the contract's content (e.g. contract value and product description) and details of the contracting process (e.g. contract award date or the procedure followed). In addition, it also contains information on the development aid projects of the contracts (e.g. project title and value). The data has wide reuse potential for researchers looking for detailed micro-level information on how major development aid spending takes place and what impacts it has. This database underlies the research article “Anti-corruption in aid-funded procurement: Is corruption reduced or merely displaced?” [1] which develops corruption risk indicators using the dataset presented.

## Specifications Table

**Table:** 

Subject	Management, Monitoring, Policy and Law
Specific subject area	Public Policy, Open Data, Development Aid, Public Procurement
Type of data	Tables
How data were acquired	Data were scraped and downloaded from the official websites of the multilateral institutions.
Data format	Raw and analyzed
Description of data collection	Scraping and downloading data involved the collection of all publicly available information related to all development aid projects as well as the corresponding procurement processes from the organisations’ official publication websites (as of 2019). All relevant fields available on the sources have been automatically collected and manually verified. Project-level data were linked to contract-level information through a unique project identifier. The list of variables was standardized and harmonized among the three multilateral development agencies. Whenever possible, values have also been standardized, for example contract values exchanged to Intl. USD. Based on the combined and standardized dataset, a list of tendering risk indicators has been developed.
Data source location	Primary data sources: the raw data on the development aid projects and corresponding procurement processes are available on the organizations’ official publication websites: • World Bank: https://projects.worldbank.org, https://finances.worldbank.org/ • Inter-Amercian Development Bank: https://www.iadb.org/en/iadb_projects/, https://www.iadb.org/en/procurement-notices-search • EuropeAid: https://ted.europa.eu/
Data accessibility	Repository name: Mendeley Data Data identification number: https://doi.org/10.17632/5mb3j5953f.1 Direct URL to data: https://data.mendeley.com/datasets/5mb3j5953f/1
Related research article	E. Dávid-Barrett, M. Fazekas, Anti-corruption in aid-funded procurement: Is corruption reduced or merely displaced?, World Development. 132 (2020) 105000. https://doi.org/10.1016/j.worlddev.2020.105000, [1].

## Value of the Data

• The exceptionally broad scope of the dataset makes it valuable for a wide set of researchers and policy analysis. It offers detailed and accurate insights into where and how development aid is spent.
• Academics, national governments, and donor agencies can use the data to monitor and assess public procurement and project performance across the world, including tracking corruption risks.
• Aid contracts and projects data can be combined with further datasets such as company registry data or sectoral performance indicators in order to gain a more comprehensive assessment of development aid effectiveness.
• This dataset adds value to existing macro-level datasets on development aid flows by providing rich micro-level information covering 3 large donors active across the globe. Micro-level data on the process and outputs of aid projects provide a much needed detail to understanding the mechanisms and constraints of facilitating development in Low and Middle Income Countries.

## 1 Data Description

The financial monitoring of the distribution of development aid has been increasingly challenging in the field of development economics and public policy. In order to move towards increased accountability and higher effectiveness of development aid, donors seek opportunities to strengthen the evidence-based and apply risk assessment models. The data presented here combines information on development aid projects of the world's largest multilateral development agencies: World Bank (WB), Inter-American Development Bank (IADB), and EuropeAid (EC). The data on projects is also linked to procurement contracts related to the implementation of these projects with a set of contract-level corruption risk indicators. The data provide a comprehensive overview of development aid spending along with a range of process and output features, also including corruption risk indicators. However, it is necessary to highlight that the datasets do not represent the full amount of development aid provided by the three donors. Due to the country-specific regulations of the development aid agencies, contracts below a certain threshold do not get published on a donor's website.

Aid-funded public procurement processes start with a call for tenders or request for quotations. This is when the buyer approaches the market or potential suppliers directly. Then, interested bidders submit their bids which are assessed by the assessment committee of the buyer. The decision is published in a contract award notification and then contract implementation commences. The procurement process is completed by delivering according to the contract or incomplete termination of the contract. Each procurement tender is part of a development aid project which are approved both by the donor and the recipient government. Typically, one project would lead to a number of procurement tenders and contracts. While these processes are complex (multi-stage, multi-level), our database contains information on major steps and features for both projects and contracts. The level of observation in the dataset is a public procurement contract which is the lowest unit of observation of the project and procurement cycles. By implication, features characterising higher-level observations such as projects are repeated for all corresponding contracts (rows).

The below data description reports parameters on an unfiltered dataset which includes all available procurement information on contracts that were both successfully concluded as well as failed/got canceled. It is also possible to select contacts from completed procedures using the condition filter_ok=1 (while there is no definitive flag on the official publication about cancellation, we denote contracts as cancelled if they fail to have a winning supplier name).

**Fig. 1:**
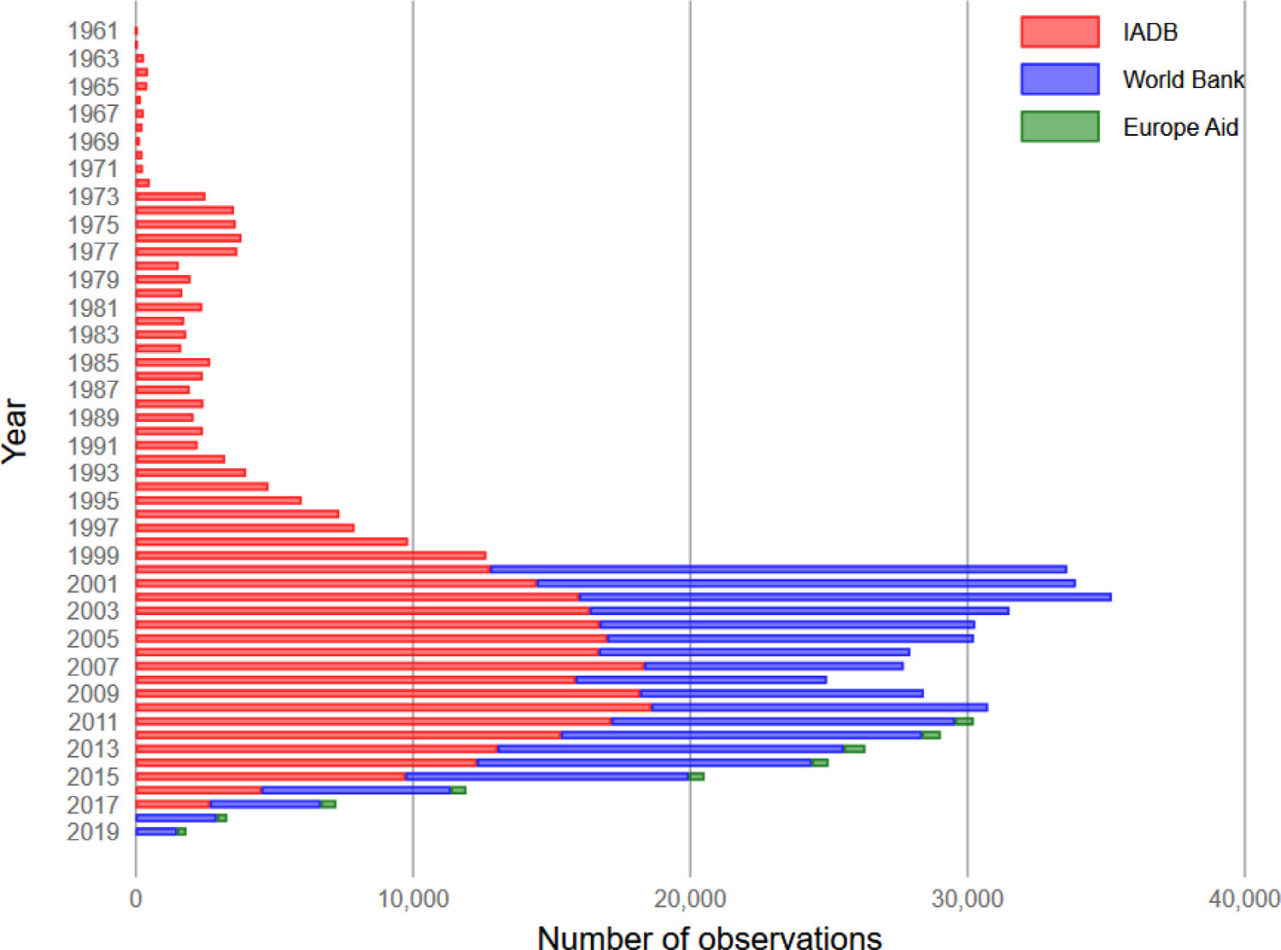
Number of observations in the dataset over 1961-2019^1^.

^1^Data for the year 2019 is incomplete.

**Fig. 2:**
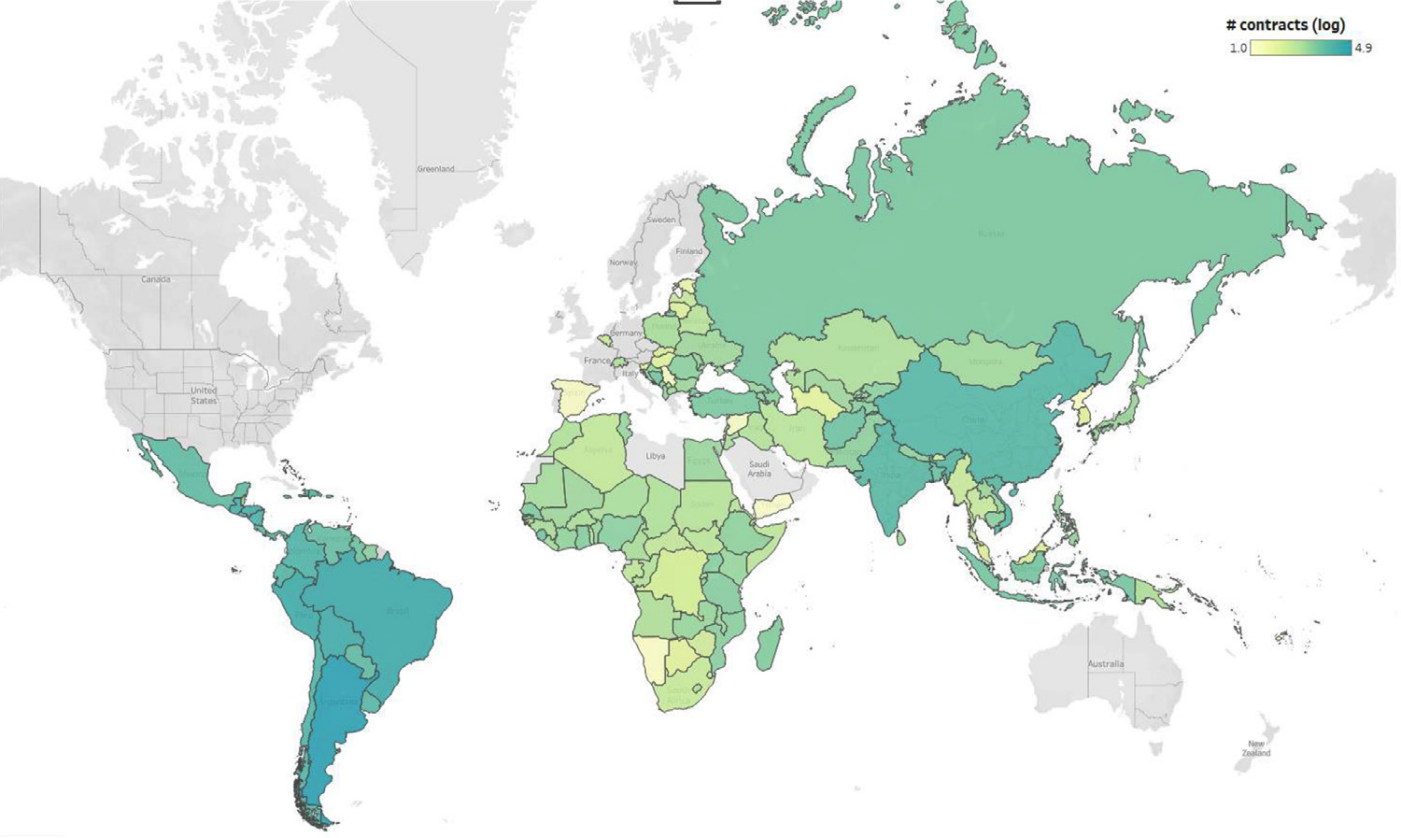
Country scope of the data, number of observations represented by colour.

**Fig. 3:**
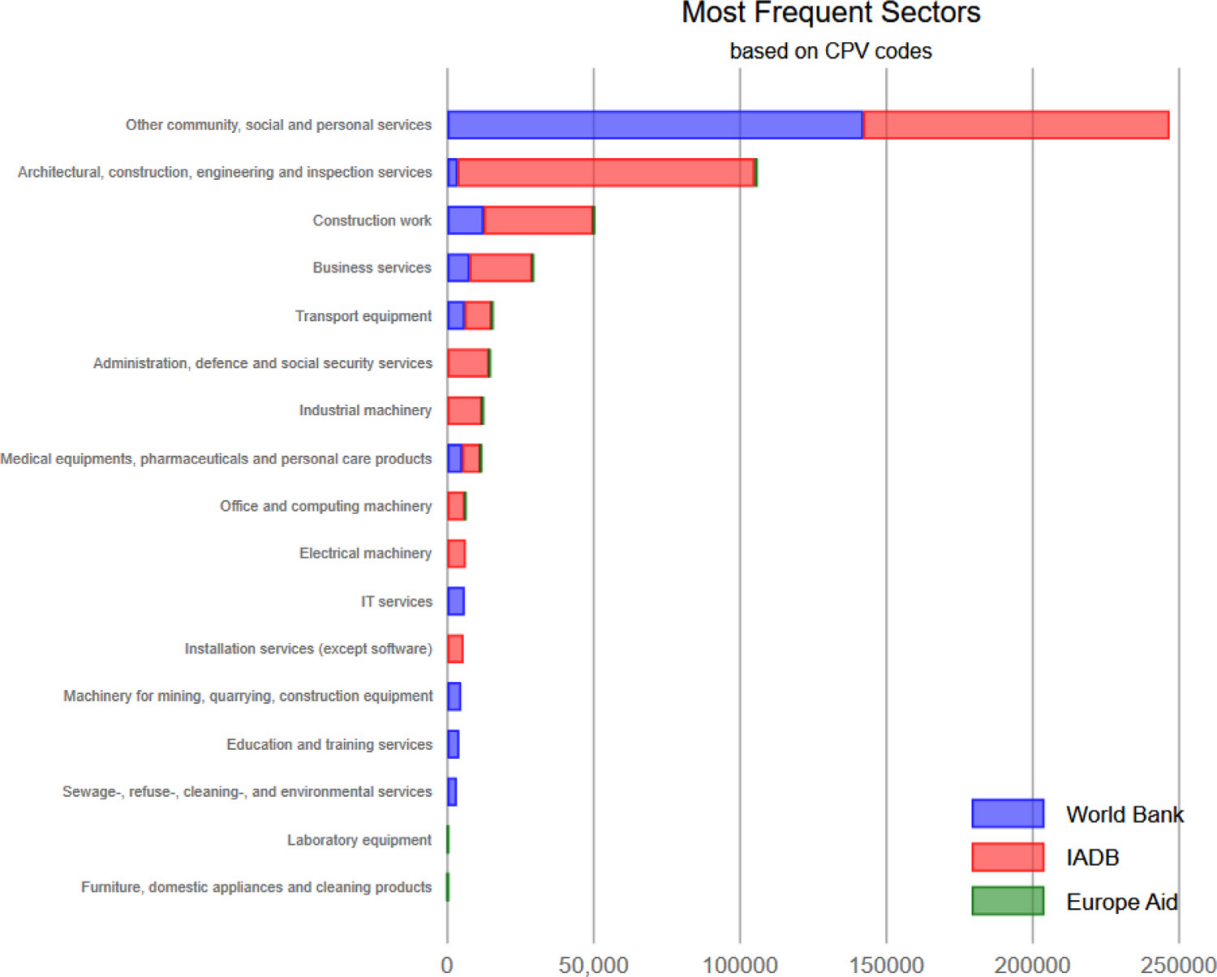
Distribution of 10 largest sectors (based on CPV codes)^1^.

^1^CPV (Common Procurement Vocabulary) codes represent a hierarchical and ordered classification of products purchased. For more information see: https://ec.europa.eu/growth/single-market/public-procurement/digital-procurement/common-procurement-vocabulary_en

The combined dataset represents more than 15,000 projects and 400,000 contracts (Table 1), covering nearly all countries of the world (Fig. 2) (there is no project information available for the EuropeAid dataset). While the IADB data goes back to 1961, the bulk of the dataset covers 2000-2017 (Fig. 1). Nevertheless, there is a notable difference between the datasets with the World Bank data being the most comprehensive. The combined dataset is also highly diverse in terms of types of products purchased ranging from social services to major construction projects (Fig. 3).

**Table 1:** Data description.

		Multilateral development agencies		
Variable		World Bank (WB)	Inter-American Development Bank (IADB)	EuropeAid (EC)
Publications	Projects	7,940	7,939	No information on projects
	Contract Notices*	34,260	No contract notices	2,714
	Contract awards	261,656	142,777	2,417
Number of public buyers		6,244	678	No data on buyers funds go to countries
Number of suppliers		95,087	221,926	1,302
Years covered by the dataset		2000 - 2019	1961 - 2017	2011 - 2019
Number of countries		176	26	151
Aggregated contract value (USD PPP)		584 Billion	177 Billion	1.22 Billion

⁎ Includes prior information notices.

## 2 Experimental Design, Materials and Methods

### 2.1 Data collection, cleaning, and standardization

The data collection process consists of a series of steps. First, we scraped and downloaded all the relevant information available on the online publication pages of the 3 agencies (World Bank, IADB, and EuropeAid). Second, we parsed, cleaned, and merged all the acquired data for the three agencies separately. Finally, we standardized and harmonized variable content, format, and measurement units across contracts coming from the 3 organisations which allowed us to construct a combined database. To provide greater detail, we discuss each step of the data collection process below. Given the heterogeneity of the 3 data sources, we report the parsing and processing steps for each multilateral agency separately.

For the World Bank dataset, the main source was the organization's website^1^. On its website, the World Bank reports the information on development aid projects as well as related contract notices, contract awards, and concluded contracts (Table 2, Panel A). We parsed the data on both projects and the associated procurement documents. The linking of the datasets was done through the unique project identification number assigned to each development aid project as well as specified in all related procurement documents. In addition to the project ID number, procurement documents have unique identifiers that allow us to link information on the contract level (Table 2, Panel B). Unfortunately, not all procurement records could be mapped to projects due to errors and inconsistencies in the source data.

**Table 2:** Data on development aid projects and associated procurement records parsed from the World Bank's website.

*Panel A*				
Raw data				
Data source name	Data source link	Years covered	Number of observations	Level of observation / unique identifier
World Bank Projects & Operations	https://projects.worldbank.org/	2000-2019	16,000	Project-level with project ID
Contract notices	https://projects.worldbank.org/en/projects-operations/procurement?lang=en&srce=both	2000-2019	36,917	Contract-level with project ID and WB notice number
Major contract awards^1^	https://finances.worldbank.org/Procurement/Major-Contract-Awards/kdui-wcs3/data	2000-2019	131,860	Contract-level with project ID and WB contract number
Contracts	https://projects.worldbank.org/en/projects-operations/procurement?lang=en&srce=both	2000-2019	142,533	Contract-level with project ID and WB contract number

*Panel B*				

Merged data				

Sources used		Years covered	Number of observations	Level of observation / unique identifier

World Bank Projects & Operations, Contract notices, Major contract awards, Contracts		2000-2019	295,916	Contract-level with project ID and WB notice number

1 The term “major contract awards” applies to contracts with value above country-specific thresholds. These contracts go through a prior review process by the World Bank implying greater central scrutiny as well as better data quality. Below-threshold contracts are fully managed by the recipients of the development aid and are not included in the dataset.

The IADB data was scraped from the organization's website. The data inputs were development aid projects information and associated contract notices and awards (Table 3, Panel A). In the process of matching the three inputs, we had to exclude contract notices since they are missing a unique identifier that could link them to contract awards. The merged dataset included project data and related contract awards that were linked through a unique project ID (Table 3, Panel B).

**Table 3:** Data on development aid projects and associated procurement records parsed from the IADB's website.

*Panel A*				
Raw data				
Data source name	Data source link	Years covered	Number of observations	Level of observation / unique identifier
Project details	https://www.iadb.org/en/projects-search?country=&sector=&status=&query=&projectTypeCombo=&fund=&finCurrency=&yearFrom=&yearTo=&financialProd=&ESIC=&financingOver=&financingUnder=&projectNumber=	1960-2017	20,905	Project level with project ID
Procurement notices	https://www.iadb.org/en/procurement-notices-search	1999-2017	15,441	Tender-level with project ID
Contract awards	https://www.iadb.org/en/iadb_projects/form/search_awarded_contracts	1961-2017	357,932	Tender-level with contract reference number and operation number

*Panel B*				

Merged data				

Sources used		Years covered	Number of observations	Level of observation / unique identifier

Project details with associated contract awards		1961-2017	391,668	Tender-level with project ID and tender source ID

In the case of the EuropeAid data, the organization's website contained limited information on projects and related procurement procedures, therefore, we used an alternative official source for data scraping – Tender Electronic Daily (TED) (Table 4, Panel A). TED is the European public platform dedicated to public procurement which publishes documentation on opportunities for public procurement as well as concluded public procurement contracts in the European Union and European Economic Area. While TED functions as the EU-wide platform, for this database compilation, we narrowed down the search of procurement records to external aid programmes and further to European Development Fund and External aid. From the TED website, we scraped all relevant contract notices and contract awards. The matching of contract notices to contract awards was done by using a combination of unique identifiers: tender ID, record iD, and lot title which enabled the identification of each lot within a tender (since there can be several lots in one contract notice). There was no project information available on this source.

**Table 4:** Data on development aid public procurement contracts funded by EuropeAid.

*Panel A*				
Raw data				
Data source name	Data source link	Years covered	Number of observations	Level of observation / unique identifier
Contract notices	http://ted.europa.eu/TED/search/search.do Selecting “External aid programmes” and “European Development Fund and External aid” from the dropdown menu of European Institutions in the advanced search.	2011-2019	5,856	Lot-level tender ID, document ID and lot title
Contract awards	http://ted.europa.eu/TED/search/search.do Selecting “External aid programmes” and “European Development Fund and External aid” from the dropdown menu of European Institutions in the advanced search.	2011-2019	1,806	Lot-level tender ID, document ID and lot title
*Panel B*				

Merged data				

Sources used		Years covered	Number of observations	Level of observation / unique identifier

Contract notices and awards		2011-2019	4,351	Lot-level tender source ID and call for tender source ID

Once the individual organizations’ data sources were scraped and merged, we standardized variable names and formats to compile the 3 datasets into a single database. Table 5 presents the list of the project- and procurement-related variables that are present in the combined dataset. As the 3 sources contain a wide set of, often idiosyncratic variables, we selected those for the combined dataset which fulfilled the following criteria:

• high value-added to the understanding of development aid projects and procurement processes
• high quality of the data and
• presence in at least two out of the three data sources.

**Table 5:** List of variables in the combined dataset and their availability depending on the source.

Variable	Variable Description	Variablec type	Variable Name in a Combined Dataset	WB	IADB	EuropeAid
**SOURCE INFORMATION**						
Donor agency	Donor agency	string	dataset	✔	✔	✔
Filter: non-missing bidder name	Filters records to non-missing supplier name	binary	filter_ok	✔	✔	✔
Procurement document type	Type of notice	string	noticetype	✔		✔
Source link	Source (url)	string	url			✔
**PROJECT VARIABLES**						
**PARAMETERS/ID**						
Project id	Project identification code	string	pr_id	✔	✔	
Project name	Project name	string	pr_name	✔		
Project description	Project description	string	pr_description	✔	✔	
Project country	Project country name	string	pr_country_name	✔	✔	
Project country (ISO ALPHA-2)	Project country (ISO 3166-1 Alpha-2 code)	string	pr_country_iso	✔	✔	
**DATES**						
Project approval date	Project approval date	string (YMD)	pr_apprdate	✔	✔	
Project closing date	Project close date	string (YMD)	pr_closedate	✔		
**TENDER/LOT LEVEL**						
**ID**						
Tender id	Tender source identification code	string	tender_sourceid	✔	✔	✔
Call for tender title	Call for tender title	string	cft_title	✔		✔
Call for tender source id	Call for tender source identification code	string	cft_sourceid	✔		✔
Contract title	Contract title	string	ca_title	✔		✔
Number of lots	Number of lots in tender	numeric	nr_lots	✔		✔
**DATES**						
Year	Year of record	numeric	year	✔	✔	✔
Call for tender publication date	Call for tender publication date	string (YMD)	cft_publdate	✔		✔
Bidding deadline	Bid deadline (from call for tender notice)	string (YMD)	ca_signdate	✔		✔
Signature date or award decision date^1^	Award date (from the contract award notice)	string (YMD)	cft_bid_deadline	✔	✔	✔
**PARAMETERS**						
Procedure type	Procedure type	string	procedure_type	✔	✔	✔
Contract type	Contract type (detailed)	string	contract_type	✔	✔	✔
Supply type	Supply type (Goods/ Works/ Services)	string	supply_type	✔	✔	
Sector	Contract sector	string	contract_sector	✔	✔	
CPV code	CPV code (CPV2008)	string	cpv_code	✔	✔	✔
**BUYER VARIABLES**						
Buyer id	Borrower identification code (GTI)	string	borrower_masterid	✔	✔	
Buyer country	Borrower country name	string	borrower_country_name	✔	✔	✔
Buyer country (ISO ALPHA-2)	Borrower country (ISO 3166-1 Alpha-2 code)	string	borrower_country_iso	✔	✔	✔
Buyer Name	Borrower name	string	borrower_name	✔	✔	
Buyer address	Borrower address	string	borrower_address	✔		✔
**BIDDER VARIABLES**						
Bidder id	Supplier identification code (GTI)	string	supplier_masterid	✔	✔	✔
Bidder country	Supplier country name	string	supplier_country_name	✔	✔	✔
Bidder country (ISO ALPHA-2)	Supplier country (ISO 3166-1 Alpha-2 code)	string	supplier_country_iso	✔	✔	✔
Bidder name	Supplier name	string	supplier_name	✔	✔	✔
Bidder address	Supplier country address	string	supplier_address		✔	✔
Number of submitted bids	Number of bids	numeric	bids_count	✔		✔
**PRICE VARIABLES**						
Contract award price (original currency)	Scraped value of contract	numeric	lot_value_reported	✔	✔	✔
Contract award currency	Currency of contract value	numeric	lot_value_reported_currency			✔
Contract award price (USD PPP)	Value of lot (Int. USD - inflation adjusted)	numeric	lot_value_usd	✔	✔	✔
**SUPPLEMENTARY VARIABLES FOR RISK INDICATOR CALCULATIONS**						
Secrecy score	Secrecy score (Financial Secrecy Index)	numeric	sec_score	✔	✔	✔
Foreign Supplier	Supplier is foreign (GTI)	numeric	fsuppl	✔	✔	✔
PPP conversion factor	GDP, PPP (current international $) [data.worldbank.org]	numeric	ppp		✔	✔
Submission period	Submission period (GTI)	numeric	submission_period	✔		✔
Decision period	Decision period (GTI)	numeric	decision_period	✔		✔
MAD (Mean Absolute Deviation as per Benford's law) value	Mean Absolute Deviation - Benford's (GTI)	numeric	MAD	✔	✔	
MAD conformity category	Conformity to Benford's law (GTI)	string	MAD_conformitiy	✔	✔	

1 Due to the lack of signature dates in the EuropeAid source, we report contract award decision dates as contract signature dates.

Overall, the shortlisted variables comprehensively describe development aid projects and the procurement processes associated with their implementation. The share of missing observations for each variable is presented in the Appendix, Table A1.

Following the harmonization of the variables’ names and formats, we performed cleaning and standardization steps to ensure the consistency of the combined data. Firstly, we created a filter (filter_ok) that narrows down the sample of procurement processes to successfully completed procedures. Due to data complexity, no criterion directly shows if an observation represents an awarded contract. Therefore, we assumed that an awarded contract has a non-missing winning supplier name, conversely, a procurement record without supplier name was not awarded. In this paper, the reported numbers represent the characteristics of the whole sample which includes both failed and completed procurement procedures. The filter variable is included in the combined dataset making filtering options easily accessible by data users.

Locations are of crucial value for a range of uses of this dataset, hence we implemented a series of data enrichment procedures. We used Here Maps API^2^ to enhance the unstructured supplier address data in the IADB dataset. We also used the “kountry” Stata module [2] to standardize all country names for projects, buyers and suppliers. As for contract values, we provided the user with the directly reported prices and the purchasing power parity adjusted prices along with the Worldbank's Purchasing power parity (PPP) conversion rates^3^. Furthermore, we enhanced the product classification for contracts without product codes. We applied a token-based string matching technique to match contracts without product codes to the Common Procurement Vocabulary (CPV 2008)^4^ based on tender/lot descriptions. Additionally, we supplemented entries with missing contract sectors using the CPV divisions from the product codes. Finally, to ensure completeness, we merged both the borrowing body and the procuring entity to generate the buyer name for the World Bank source while it is generated only using the procuring entity name in the IADB source.

### 2.2 Calculated risk indicators

**Fig. 4:**
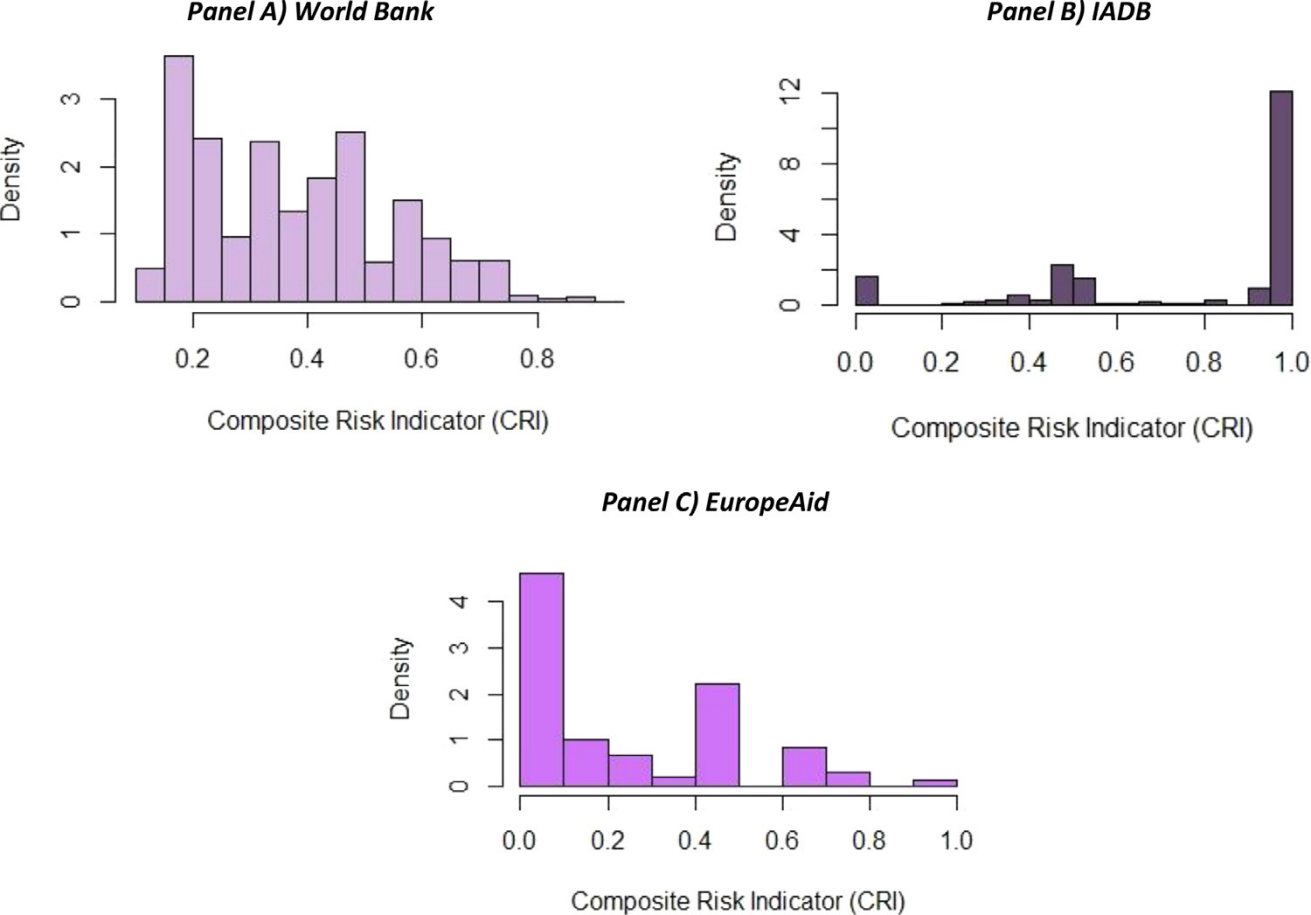
Distribution of Composite Risk Indicator (CRI) by data source.

Given that risk assessment is a major use case for the dataset, a set of risk indicators have been calculated based on the available project and procurement data. These corruption risk indicators capture the restricted and unfair access to public resources benefiting connected bidders in public procurement [3]. Risk indicator development and validation are based on already established methodologies [4]. Some of these risk indicators are also used in the linked publication for this article [1]. All risk indicators are calculated at the contract level, their summary and availability by data source are presented in Table 6, while Fig. 4 presents the composite risk indicator, CRI for each source.

**Table 6:** Procurement risk indicators summary.

Indicators			Availability by Data Source		
Indicator	Indicator Description	Indicator Name in a Combined Dataset	WB	IADB	EuropeAid
Single bidding	0 = more than one bid received 1 = one bid received	singleb	✔		✔
Procedure type	0 = open or low-risk procedure types 1 = moderate-risk procedure type 2 = high-risk procedure types 99 = missing procedure type	corr_proc	✔	✔	✔
No call for tender	0 = call for tenders advertised 1 = call for tenders not advertised	nocft	✔		✔
Submission period length	Categorized according to a risk level length of a period between publication of call for tenders and submission deadline: 0 = low-risk submission period length range 1 = high-risk submission period length range 99 = missing submission period	corr_submp	✔		✔
Decision period length	Categorized according to a risk level length of a period between submission deadline and announcing contract award: 0 = low-risk decision period length range 1 = high-risk decision period length range 99 = missing decision period	corr_decp	✔		✔
Buyer spending concentration	Share of contract value won by the largest supplier in the total annual spending of a buyer, %	proa_ycsh	✔	✔	
Buyer share of consultancy spending	Categorized according to a risk level share of consultancy spending in the total spending of a buyer: 0 = low-risk share of consultancy spending 1 = high-risk share of consultancy spending	corr_cons	✔	✔	
Benford's law [5]	Categorized according to a risk level MAD values: 0 = low-risk MAD range 1 = moderate-risk MAD range 2 = high-risk MAD range 99 = not enough observations to calculate MAD	corr_benford	✔	✔	
Tax haven indicator [6]	0 = supplier is not registered in a high financial secrecy jurisdiction 1 = supplier is registered in a high financial secrecy jurisdiction 99 = supplier country information is not available	taxhav	✔	✔	✔
Agency capture	Risk category assigned according to the share of the largest supplier in the buyer's total annual spending: 0 = less than 50% supplier share 1 = more than 50% supplier share	proa_capt50	✔	✔	
Corruption Risk Index (CRI)	Composite corruption risk score. It is the arithmetic average of valid and non-missing individual corruption risk indicators.	cri	✔	✔	✔

While this article presents an extensive description of available data and constructed individual as well as composite corruption risk scores, it does not present an exhaustive list of potential data applications. In addition to monitoring and assessing corruption risks in public procurement related to development aid projects, the dataset introduces the opportunity to measure transparency in development project documentation by inspecting what kind of data is available and what crucial pieces of information are missing. Missing bits of information in tender documentation could potentially be a result of a deliberate action aimed at limiting public access to some crucial facts such as, for instance, bidder name, title, contract value, procurement method, etc. Furthermore, the compiled database offers a great potential for further competition and collusion research given a wide pool of contracts and a high level of data granularity. With the available data, it is possible to shift a level of observation from a single contract to a more aggregated level of a bidder, buyer, product market, country, etc., to observe participants’ behavior and the dynamics of market structure.

## Ethics Statement

The data were obtained from the official websites of the World Bank, Inter-American Development Bank, and EuropeAid which publish the data with the aim of providing transparency and supporting accountability of their operations and spending. The data includes information on organisations and formal tenders and contracts, hence do not fall under personal data protection regulations in Europe or elsewhere (i.e. no personal information is processed).

## CReDiT Author Statement

**Mihály Fazekas:** Conceptualization, Formal analysis, Funding acquisition, Investigation, Methodology, Project administration, Resources, Software, Supervision, Writing – original draft, Writing – review & editing; **Aly Abdou:** Data curation, Investigation, Methodology, Software, Validation, Visualization, Writing – original draft; **Yuliia Kazmina:** Data curation, Investigation, Methodology, Software, Validation, Visualization, Writing – original draft; **Nóra Regős:** Data curation, Investigation, Methodology, Software, Validation, Visualization.

## Declaration of Competing Interest

The authors declare that they have no known competing financial interests or personal relationships which have, or could be perceived to have, influenced the work reported in this article.

## Figures and Tables

**Table A1:** Report on missing values.

Variable	# of Missing Observations	Share of Missing Observations, %
admin_capacity	102204	14.77
bids_count	566770	81.91
borrower_address	648734	93.76
borrower_counrty_name	45223	6.54
borrower_country_iso	60662	8.77
borrower_masterid	338489	48.92
borrower_name	346320	50.05
ca_signdate	108503	15.68
ca_title	458977	66.33
cft_bid_deadline	651726	94.19
cft_publdate	616923	89.16
cft_sourceid	616923	89.16
cft_title	616923	89.16
contract_sector	100873	14.58
contract_type	102445	14.81
corr_benford	4351	0.63
corr_cons	35404	5.12
corr_decp	391668	56.6
corr_proc	287686	41.58
corr_submp	391668	56.6
cpv_code	0	0
cri	69557	10.05
dataset	0	0
decision_period	686660	99.24
filter_ok	0	0
fsuppl	128180	18.52
lot_value_reported	102065	14.75
lot_value_reported_currency	462680	66.87
lot_value_usd	157872	22.82
MAD	429917	62.13
MAD_conformity	429917	62.13
nocft	393593	56.88
noticetype	391672	56.61
nr_lots	463075	66.92
ppp	382780	55.32
pr_apprdate	408917	59.1
pr_closedate	422938	61.12
pr_country_iso	344223	49.75
pr_country_name	332102	48
pr_description	580457	83.89
pr_id	327976	47.4
pr_name	396019	57.23
proa_capt100	370848	53.6
proa_capt50	425535	61.5
proa_ycsh	405941	58.67
procedure_type	369864	53.45
sec_score	456470	65.97
singleb	566941	81.94
source	0	0
submission_period	652997	94.37
supplier_address	345145	49.88
supplier_country_iso	103069	14.9
supplier_country_name	102803	14.86
supplier_masterid	69557	10.05
supplier_name	69557	10.05
supply_type	98090	14.18
taxhav	0	0
tender_sourceid	75858	10.96
url	687584	99.37
year	29965	4.33

**Table A2:** Validation results.

## Data Availability

Development Aid Contracts Database (Original data) (Mendeley Data).

## Appendix

Tables A1 and A2

## Footnotes

1 https://www.worldbank.org/

2 https://developer.here.com

3 https://data.worldbank.org/indicator/PA.NUS.PPP

4 We add 3 product codes for unclassified contracts 99100000 for unclassified supplies, 99200000 for unclassified services, 99300000 for unclassified works, and 99000000 for contracts with missing supply types.
